# Influence of *Helicobacter pylori* eradication blister-pack handling on outpatient AWaRe access share: a Japanese community pharmacy-based sensitivity analysis

**DOI:** 10.1186/s40780-026-00594-y

**Published:** 2026-06-12

**Authors:** Yuki Kishino, Yoshiharu Fukuda, Shinya Kiyokawa, Kaei Hiroi

**Affiliations:** 1 PHARUMO, Inc., 52nd Floor, Tokyo Opera City Tower 3-20-2 Nishi-Shinjuku, Shinjuku-ku, Tokyo, 163-1452 Japan; 2 Teikyo University, Tokyo, Japan

**Keywords:** AWaRe, *Helicobacter pylori*, Access share, Defined daily dose, Days of therapy, Community pharmacy, Dispensing data, Pharmacoepidemiology

## Abstract

**Background:**

*Helicobacter pylori* (*H. pylori*) eradication regimens may influence outpatient AWaRe Access share when routine surveillance is based on oral antibacterials classified under the Anatomical Therapeutic Chemical (ATC) code J01. This study evaluated how commercially identifiable *H. pylori* eradication blister-pack handling affects the reported AWaRe Access share using community pharmacy dispensing data.

**Methods:**

We analyzed monthly outpatient dispensing data for oral antibacterials in ATC J01 from community pharmacies in Nayoro, Japan, from July 2022 to March 2025. AWaRe Access share was primarily compared between an unadjusted series and a pack-adjusted series in which commercially identifiable eradication blister packs were handled using a predefined pack-derived offset. An exploratory extended adjustment also considered probable non-pack eradication regimens. Blister packs were identified by product name. In the pack-adjusted series, pack days of therapy were conservatively converted into a DDD-equivalent offset and subtracted from both the numerator and denominator. The primary outcome was the monthly difference in Access share between the unadjusted and pack-adjusted series (ΔAccess).

**Results:**

Among 20,129 oral J01 records, commercially identifiable eradication blister packs accounted for a median of 1.13% of monthly records and 0.55% of monthly DOT. The difference between the unadjusted and pack-adjusted series (ΔAccess) was positive in all 33 months, with a median of 0.59% points (pp) (IQR, 0.47–0.83; range, 0.26–1.43). These findings indicate that the handling of commercially identifiable eradication blister packs had a consistent but modest influence on the reported Access share.

**Conclusions:**

The handling of commercially identifiable *H. pylori* eradication blister packs had a consistent but modest influence on outpatient AWaRe Access share derived from dispensing data. Although the observed difference was modest in this low-Access-share setting, explicit reporting of blister-pack handling may improve transparency, particularly in benchmark-based or close comparative evaluations. Routine AWaRe surveillance should therefore define how commercially identifiable eradication packs are handled, ideally using standardized adjustment or dual reporting.

## Background

Antimicrobial resistance (AMR) is a major global challenge across clinical medicine, public health, and economic systems, and antimicrobial stewardship (AMS) remains central to efforts to address it [1]. The World Health Organization (WHO) AWaRe (Access, Watch, Reserve) classification has been widely adopted as a framework for monitoring antibiotic use, including in outpatient settings, and targets such as an Access share of at least 60% have been used as benchmarks for interpreting prescribing quality [2]. Routine surveillance commonly relies on standardized quantitative measures such as the defined daily dose (DDD), allowing antibiotic use to be aggregated and compared across settings [3].

However, interpretation of AWaRe-based indicators may be affected by clinical contexts in which treatment regimens include both systemic antibacterials and non-antibacterial agents. In Japan, *Helicobacter pylori* eradication therapy represents one such context. Eradication therapy has been widely implemented under the national insurance system and was further expanded to chronic gastritis in 2013 [4–7]. Standard first-line treatment consists of a 7-day triple regimen including amoxicillin (AMPC; Access), clarithromycin (CAM; Watch), and a proton pump inhibitor (PPI; A02BC, outside ATC J01) [8]. For routine practice, commercially identifiable eradication blister packs such as VONOSAP^®^ and VONOPION^®^ are widely available [9–11].

Because routine AWaRe reporting for outpatient antibiotic use is typically based on systemic antibacterials classified under ATC J01, such regimens may affect the Access numerator and the J01 denominator asymmetrically. AMPC contributes to the Access numerator, whereas the PPI component does not contribute to the J01 denominator, which may lead to a higher reported Access share depending on how such regimens are handled [2, 3]. In other words, when eradication regimens contain both J01 antibacterials and non-J01 agents, routine J01-based aggregation may attribute the antibacterial component to the reported Access share while leaving the non-J01 component outside the denominator, thereby affecting the reported Access share.

In Japanese antimicrobial surveillance practice, *H. pylori* eradication regimens are operationally handled separately from routine oral antibacterial surveillance categories. National surveillance materials classify *H. pylori* eradication drugs as a distinct operational category and apply separate DDD-processing rules for these regimens [12]. However, the quantitative impact of this handling on AWaRe-based outpatient indicators has not been well characterized, and it remains unclear to what extent such handling is consistently reflected in real-world dispensing-based surveillance datasets.

In this study, we used dispensing data from community pharmacies in Nayoro City, Hokkaido, Japan, as a case study to evaluate how blister-pack handling influences the reported AWaRe Access share and to assess a practical pack-adjusted sensitivity analysis. Nayoro is a centralized rural setting in which one hospital accounts for a large proportion of prescriptions [13], providing a useful setting for examining the impact of eradication regimens on AWaRe Access share. Using 33 months of outpatient dispensing data, we compared unadjusted reporting with a pack-adjusted series based on commercially identifiable eradication blister packs and conducted an exploratory extended adjustment including probable non-pack eradication regimens. Our objective was to evaluate the influence of commercially identifiable *H. pylori* eradication blister-pack handling on routine AWaRe Access-share reporting using community pharmacy dispensing data.

## Methods

### Study design, setting, and data source

We conducted a retrospective observational study using anonymized dispensing data from seven community pharmacies in Nayoro City, Hokkaido, Japan, from July 2022 to March 2025. The participating pharmacies represented 58% of all community pharmacies in the city and captured approximately 69.6% of community-dispensed outpatient prescriptions during the study period, based on local pharmacy association data reported in our previous regional analysis [13]. In that analysis, Nayoro was described as a relatively centralized prescribing environment, with a large proportion of outpatient prescriptions originating from Nayoro City General Hospital and broadly similar prescribing trends observed across participating pharmacies. Therefore, the dataset was used to assess regional outpatient prescribing trends, although it did not represent complete capture of all prescriptions in the city.

The dataset comprised two linked components: (1) raw dispensing records containing product name, dispensing month, days of therapy (DOT), and quantity dispensed; and (2) an annotation table assigning each product its WHO AWaRe category (2023 classification [2]), Japanese defined daily dose (jDDD, based on the WHO ATC/DDD framework [3]), and labeled strength. Records were linked by brand name or YJ code, restricted to systemic oral antibacterials classified under ATC J01, and aggregated by dispensing month (ISO year–month format).

### Drug cohort and preprocessing

The analytical cohort included all outpatient dispensing records for systemic oral antibacterials classified under ATC J01. Antifungal agents (J02) and antiviral agents (J05) were excluded. Brand and generic names were cross-walked and normalized to unify ingredient-level labeling. Data cleaning was performed before monthly aggregation using predefined rules developed for this analysis. Records were excluded if essential fields, including dispensing month, product name, DOT, or quantity, were missing; if the product could not be mapped to an oral systemic antibacterial classified under ATC J01; or if the record represented a non-systemic antibacterial, antifungal agent, or antiviral agent. Records with DOT exceeding 90 days, zero or negative quantities, or quantities inconsistent with the labeled strength were manually reviewed against the product annotation table. When the inconsistency could be resolved by product-name normalization, YJ code mapping, or standardized labeled-strength assignment using the annotation table, the resolved value was used. Otherwise, the record was excluded from the analysis.

### DDD assignment and monthly aggregation

For each dispensing record, DDDs were calculated using labeled strength and the Japanese antimicrobial master, which includes antimicrobial-specific Japanese DDD (jDDD) values and AWaRe classifications used in Japanese antimicrobial use surveillance [12]. The jDDD values were interpreted within the WHO ATC/DDD methodological framework [3]. The Japanese antimicrobial master used in this study was the version available at the time of analysis in December 2025.

Labeled strengths were primarily obtained from the annotation table; when unavailable, they were programmatically extracted from product names (e.g., values expressed in mg, g, or %). Monthly totals of all J01 DDDs (denominator) and Access-group DDDs (numerator) were then calculated.

For co-trimoxazole (sulfamethoxazole/trimethoprim; brand names Baktar and Daifen), the jDDD is defined in unit doses (4 unit doses/day); therefore, DDDs for these products were calculated on a unit-dose basis rather than by active ingredient mass.

### Identification of *H. pylori* eradication blister packs

Commercially identifiable *H. pylori* eradication blister packs were identified by product-name matching. The search included marketed pack products for *H. pylori* eradication, including VONOSAP^®^, VONOPION^®^, RABECURE^®^, and other available eradication pack products. The main pack-adjusted analysis was restricted to pack products that were repeatedly observed during the study period and could be summarized reproducibly at the monthly level. Pack products with fewer than five records during the study period were not included in the monthly adjustment because of unstable estimation and sparse-cell confidentiality considerations.

### Outcome definitions

The primary outcome was the monthly AWaRe Access share, defined as the proportion of Access-group DDDs among all systemic oral antibacterial DDDs classified under ATC J01. Three monthly series were considered. First, the unadjusted Access share was calculated as:

Access DDD / total J01 DDD × 100.

Second, the pack-adjusted Access share was calculated by removing commercially identifiable eradication blister packs using a conservative pack-level operational adjustment. Because component-level DDD reassignment within multi-component eradication packs was not feasible in a reproducible manner, pack DOT was converted into a DDD-equivalent pack-derived offset and subtracted from both the Access numerator and the total J01 denominator:

(Access DDD − pack-derived offset) / (total J01 DDD − pack-derived offset) × 100.

This adjustment was designed to provide a conservative and operationally reproducible estimate of the direction and approximate magnitude of the indicator change associated with eradication-pack handling.

Third, ΔAccess was defined as the monthly difference between the unadjusted and pack-adjusted Access share, expressed in percentage points (pp):

ΔAccess = Access share (unadjusted) − Access share (pack-adjusted).

Secondary outcomes included the monthly prevalence of eradication packs, expressed as the proportion of all oral J01 dispensing records by record count and by DOT. DDD-based pack prevalence was not calculated because eradication packs contain both antibacterial and non-antibacterial components, making component-level DDD allocation within multi-component packs operationally ambiguous.

### Statistical analysis

The primary analysis was descriptive and comparative at the monthly level. For each month, we calculated the unadjusted Access share, pack-adjusted Access share, ΔAccess, and pack prevalence. We then summarized the distribution of ΔAccess across the 33-month observation period using medians, IQRs, and ranges.

Because the principal aim of the study was to evaluate indicator changes under different regimen-handling assumptions and to assess a practical pack-adjusted sensitivity analysis for routine surveillance, emphasis was placed on the consistency and magnitude of monthly differences rather than on formal hypothesis testing. This analysis was interpreted as a sensitivity analysis of indicator construction rather than as an estimation of deviation from an external gold-standard Access share.

### Sensitivity and exploratory analyses

We performed three pre-specified sensitivity or exploratory analyses to assess the robustness of the pack-adjusted estimates. First, the DOT-to-DDD equivalence used in the pack-derived offset was varied by ± 20%. Second, an AMPC-only offset was examined as a conservative alternative scenario. Third, we conducted an exploratory extended adjustment to examine the possible influence of probable non-pack *H. pylori* eradication regimens. Candidate regimens were defined as AMPC and CAM courses of 7 ± 2 days dispensed on the same day or on adjacent days within the same month. Because diagnostic information and systematic linkage to acid-suppressive therapy were unavailable, this algorithm was expected to have limited specificity and was used only as a supportive, hypothesis-generating sensitivity analysis.

### Statistical software

All analyses were performed using R version 4.5.1 (R Foundation for Statistical Computing, Vienna, Austria). Major packages included data.table for data manipulation, dplyr for aggregation, lubridate for date handling, sandwich for robust standard errors, lmtest for regression diagnostics, and ggplot2 for visualization.

### Missing data, data quality, and ethics

No dispensing months were missing during the observation period, and no month had a zero denominator for J01 DDD; therefore, the pre-specified rule of not computing ratios when the denominator was zero was not triggered. This study used anonymized secondary data and involved no direct participant contact. Before study initiation, the Teikyo University Research Ethics Committee was consulted and confirmed that the project fell outside the scope of formal ethics review.

## Results

### Study cohort characteristics

Over the 33-month observation period (July 2022 to March 2025), 20,129 outpatient dispensing records for systemic oral antibacterials (ATC J01) were analyzed. The median monthly record count was 643 (IQR, 588–732; range, 480–787), corresponding to a median monthly total of 5,376 J01 DDDs (IQR, 5,105–5,682; range, 3,873–6,580) and 9,147 J01 DOT (IQR, 8,624–9,790; range, 6,519–10,481) (Table 1).

**Table 1:** Summary of monthly AWaRe Access-share indicators and *H. pylori* eradication pack prevalence

Indicator	Median	IQR	Range
Monthly oral J01 dispensing records, n	643	588–732	480–787
Total J01 DDD	5,376	5,105–5,682	3,873–6,580
Total J01 DOT	9,147	8,624–9,790	6,519–10,481
Access share, unadjusted (%)	37.56	34.20–39.88	28.46–45.73
Access share, pack-adjusted (%)	36.71	33.08–39.34	27.64–45.33
ΔAccess (pp)	0.59	0.47–0.83	0.26–1.43
Blister-pack prevalence by record count (%)	1.13	0.93–1.66	0.42–2.83
Blister-pack prevalence by DOT (%)	0.55	0.45–0.76	0.21–1.28

Values are summarized across 33 monthly observations from July 2022 to March 2025. Unadjusted = no pack adjustment. Pack-adjusted = predefined pack-derived offset applied to both the Access numerator and total J01 denominator. ΔAccess = unadjusted Access share − pack-adjusted Access share. DDD = defined daily dose; DOT = days of therapy; IQR = interquartile range; pp = percentage points

According to the WHO AWaRe classification, the overall composition of the study cohort was 33.08% Access, 62.44% Watch, and 4.48% Reserve by record count, and 37.09% Access, 59.14% Watch, and 3.77% Reserve by DDD. This distribution indicates a relatively low Access share in the study setting.

### Prevalence of *H. pylori* eradication blister packs

During the 33-month observation period, 20,129 outpatient dispensing records for systemic oral antibacterials classified under ATC J01 were included in the analysis. Commercially identifiable *H. pylori* eradication blister packs included in the main pack-adjusted analysis, namely VONOSAP^®^ and VONOPION^®^, accounted for 263 records, representing 1.31% of all oral J01 dispensing records; 245 records were VONOSAP^®^ and 18 were VONOPION^®^. On a monthly basis, the median prevalence of these packs was 1.13% by record count (IQR, 0.93–1.66%; range, 0.42–2.83%) and 0.55% by DOT (IQR, 0.45–0.76%; range, 0.21–1.28%).

RABECURE^®^ was searched for but observed fewer than five times during the study period and was not included in the monthly adjustment because of sparse-cell confidentiality and unstable estimation. Other marketed eradication pack products were searched for but were not observed in the dataset.

### Monthly difference in AWaRe Access share

**Fig. 1:**
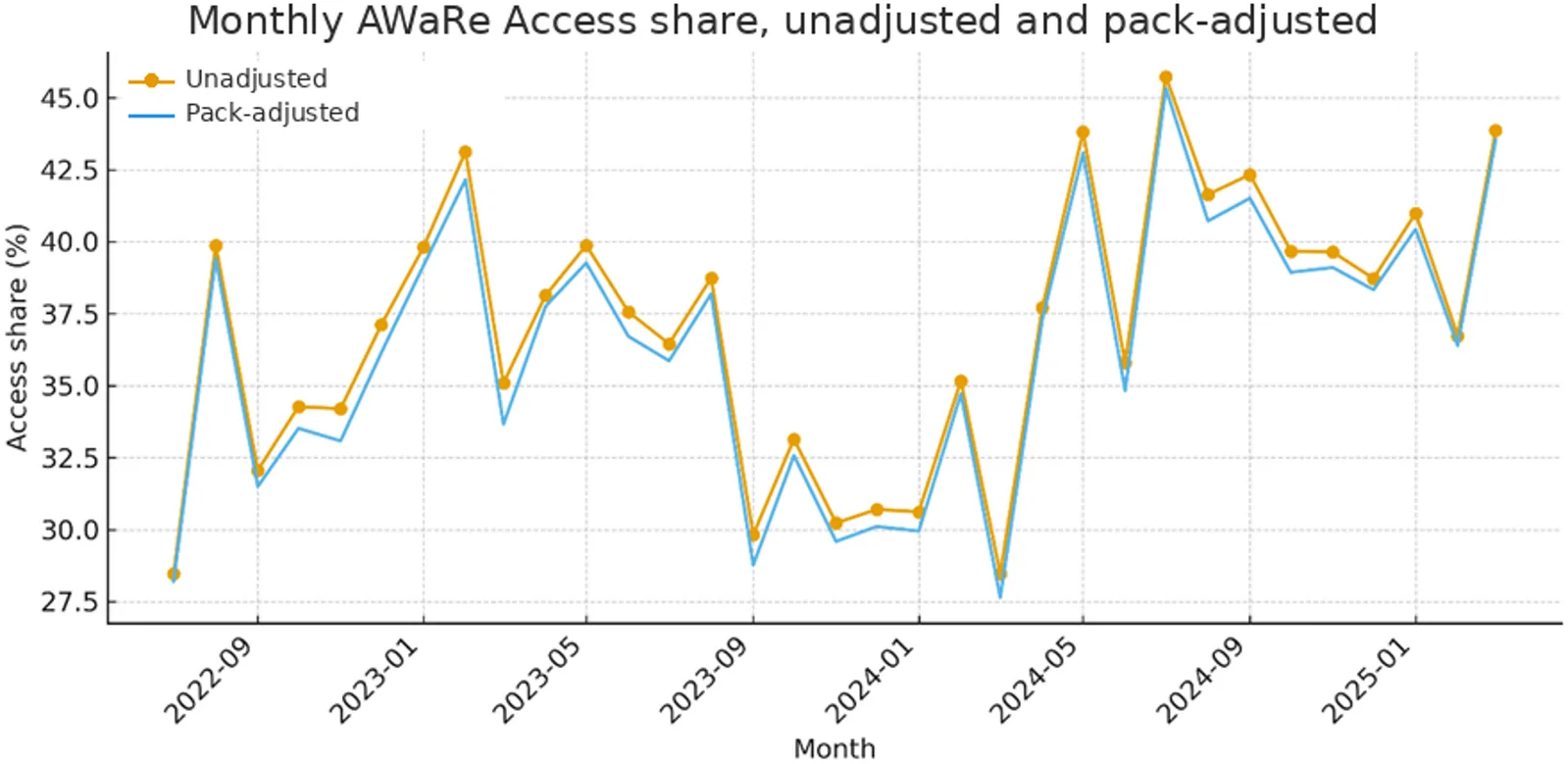
Monthly AWaRe Access share for systemic oral J01 antibacterials. The unadjusted and pack-adjusted series are shown from July 2022 to March 2025. Pack-adjusted Access share was calculated after applying the predefined pack-derived offset to both the Access numerator and total J01 denominator. Packs were identified by product-name matching. The x-axis is restricted to the study period

In the unadjusted series, the monthly AWaRe Access share ranged from 28.46% to 45.73%, with a median of 37.56%. In the pack-adjusted series, the monthly Access share ranged from 27.64% to 45.33%, with a median of 36.71%. The monthly difference between the unadjusted and pack-adjusted series (ΔAccess) was positive in all 33 months. The median ΔAccess was 0.59 pp, with a range of 0.26–1.43 pp. Thus, commercially identifiable eradication blister packs were associated with a consistently higher reported outpatient Access share, although the absolute difference was modest. Figure 1 shows the unadjusted and pack-adjusted monthly Access-share series. Table 1 summarizes the Access-share indicators, pack prevalence, and ΔAccess.

### Sensitivity and exploratory analyses

The direction and overall magnitude of the findings were consistent across all pre-specified sensitivity analyses.

First, when the DOT-to-DDD equivalence used for the pack-derived offset was varied by ± 20% relative to the baseline definition, the median ΔAccess was 0.46 pp under the − 20% scenario and 0.70 pp under the + 20% scenario, compared with 0.59 pp in the primary analysis. In both scenarios, ΔAccess remained positive in all 33 months.

Second, under the AMPC-only offset scenario, designed as a deliberately conservative alternative, the median ΔAccess was 0.204 pp (IQR, 0.156–0.337; range, 0.058–0.595), and all 33 months remained positive.

Third, in the exploratory extended adjustment, we additionally identified candidate AMPC + CAM courses of 7 ± 2 days dispensed on the same day or on adjacent days within the same month. Across the 33-month period, the total candidate DOT was 2,896 days. Under this extended adjustment, the median ΔAccess increased to 2.265 pp (IQR, 1.891–2.914; range, 1.362–3.900), while remaining positive in all months. Because this algorithm could not incorporate diagnostic information or linkage to acid-suppressive therapy, these results should be interpreted as exploratory and hypothesis-generating rather than definitive evidence of non-pack eradication-related influence.

## Discussion

### Principal findings

This study showed that the handling of commercially identifiable *H. pylori* eradication blister packs had a consistent but modest influence on outpatient AWaRe Access share when routine reporting is based on systemic antibacterials classified under ATC J01. Although eradication packs represented only a small fraction of total outpatient antibacterial use in this dataset (median 1.13% of monthly J01 records and 0.55% of monthly DOT), the difference between the unadjusted and pack-adjusted series (ΔAccess) was positive in all 33 months, with a median of 0.59 pp. The main finding of this study is therefore limited to commercially identifiable eradication blister packs. Exploratory analyses of probable non-pack regimens are discussed separately because their specificity was limited in the absence of diagnostic information.

### Interpretation and practical relevance

The AWaRe framework is intended to support interpretation of prescribing quality and to encourage greater use of Access-group agents. However, in settings where treatment regimens combine J01 antibacterials with non-J01 agents, the resulting Access share may be affected if the handling of such regimens is not explicitly defined. In the present study, the median ΔAccess of 0.59 pp was modest, and the overall Access share remained in the 30%–40% range. Therefore, the pack-adjusted analysis does not change the immediate interpretation of this region as a low-Access-share setting relative to the WHO target of at least 60%. However, a difference of approximately 0.5–1.0 pp may be relevant in benchmark-based evaluations, close comparisons across facilities or regions, or before-and-after assessments of stewardship interventions, particularly when Access shares are close to a decision threshold. These findings therefore support explicit reporting of how *H. pylori* eradication packs are handled, rather than implying that such packs substantially alter stewardship priorities in the present setting.

### Exploratory non-pack regimen analysis

The main adjustment in this study was intentionally restricted to commercially identifiable blister packs that could be reproducibly flagged by product name. In routine practice, *H. pylori* eradication therapy may also be prescribed as non-pack regimens, such as combinations of amoxicillin and clarithromycin with acid-suppressive therapy. However, because diagnostic information and systematic linkage to proton pump inhibitors were unavailable, non-pack eradication regimens could not be identified with high specificity in the present database.

The exploratory extended analysis using AMPC + CAM courses of 7 ± 2 days yielded a larger ΔAccess than the pack-only analysis. However, this finding should be interpreted only as suggesting the possibility that additional regimen-handling effects may exist beyond commercially identifiable blister packs. It should not be interpreted as a definitive estimate of the impact of non-pack eradication therapy. Future studies using diagnostic information, prescription-level linkage to acid-suppressive therapy, or validated algorithms are needed before non-pack regimen adjustment can be incorporated into routine AWaRe surveillance. Because the exploratory algorithm was based on dispensing patterns rather than confirmed diagnostic linkage, false-positive classification of non-eradication AMPC + CAM combinations cannot be excluded; accordingly, this extended analysis should be interpreted as supportive and exploratory rather than definitive.

### Methodological contribution

This study provides a simple and reproducible workflow for improving transparency in AWaRe surveillance using routine community pharmacy dispensing data. The workflow consists of three steps: (1) identifying commercially available eradication packs by product name or YJ code; (2) calculating monthly J01 DDD totals and Access-group DDD totals using labeled strengths and the Japanese DDD table (jDDD); and (3) generating an adjusted series by applying a pack-derived offset to both the numerator and denominator.

Although component-level DDD reassignment within multi-component regimens might appear theoretically preferable, it is difficult to implement consistently in routine dispensing datasets because product-level aggregation, varying dispensing practices, and incomplete linkage of non-J01 components can limit reproducibility. We therefore prioritized a pack-derived offset that could be applied transparently and consistently within routine surveillance workflows. This approach provides a conservative and implementable adjustment that is intended to preserve the direction of the effect.

### International context and the Japanese setting

Japan provides a distinctive setting for this question because *H. pylori* eradication therapy has been widely implemented under the national insurance system, and commercially identifiable eradication blister packs are used in routine practice. The magnitude of the resulting indicator change may differ across countries depending on reimbursement systems, prescribing customs, and the availability of fixed-dose eradication packs. Nevertheless, the broader methodological issue is not unique to Japan: whenever routine antibiotic indicators are derived from J01-only aggregation while clinically relevant regimens include non-J01 components, the resulting Access share may be affected by handling rules. Transparent reporting or explicit adjustment protocols may therefore be relevant in other healthcare settings as well.

### External validity and regional context

This study was conducted in Nayoro City, a relatively centralized rural setting in which a large proportion of outpatient prescriptions originated from a single core hospital and broadly similar prescribing trends were observed across participating pharmacies in our previous regional analysis. This concentration provided a coherent setting for evaluating blister-pack handling but limits generalizability. The magnitude of ΔAccess may vary in regions with different *H. pylori* screening and eradication practices, different use of commercially packaged versus non-pack regimens, more heterogeneous prescribers, or different pharmacy coverage. Therefore, the observed ΔAccess should be interpreted as a single-region estimate rather than a directly generalizable value for all Japanese outpatient settings. Community pharmacy dispensing data remain useful for regional outpatient surveillance because they reflect medications actually supplied to patients and allow unified calculation of DDD- and DOT-based indicators.

### Limitations

This study has several limitations. First, the main identification strategy was limited to major commercially identifiable eradication blister packs, and future implementation would require periodic updating as new products or generic equivalents enter the market. Second, the pack-derived offset was based on an operational DOT-to-DDD approximation rather than an exact component-level reassignment. Third, this was a retrospective analysis from a single region using community pharmacy dispensing data. Nayoro City has a relatively centralized prescribing environment, and the observed magnitude of ΔAccess may differ in urban or more heterogeneous regions, areas with different *H. pylori* eradication practices, or datasets with different pharmacy coverage. Although dispensing data reflect medications actually supplied to patients, patient-level clinical variables, diagnostic information, and demographics were unavailable. Fourth, labeled-strength extraction from product names may have introduced minor item-level uncertainty for some formulations. Finally, other clinical contexts may also affect outpatient AWaRe indicators, including specialty-specific prescribing patterns and prophylactic use, and deserve further investigation.

### Practice and policy implications

The principal practical implication of this study is that routine AWaRe surveillance should explicitly define how *H. pylori* eradication regimens are handled. Two pragmatic options are available: standardized adjustment or dual reporting of unadjusted and adjusted series. Either approach would improve interpretability and reduce the risk of misclassification in threshold-based assessments or close benchmarking exercises. Operationally, implementation is straightforward: maintain an updated product or YJ code list, flag eligible packs during routine aggregation, and apply the predefined pack-derived offset as part of standard reporting. This workflow may also be adaptable to larger administrative or claims-based surveillance datasets, provided that product-level identification and coding systems are available.

### Future work and standardization roadmap

Future studies should validate these findings in other geographic and clinical settings, refine algorithm-based identification of probable non-pack eradication regimens, and develop shared resources such as product lists and open analysis code. At a broader level, national or regional surveillance frameworks may benefit from recommending explicit handling rules or dual-series reporting so that outpatient AWaRe indicators can be interpreted more transparently and compared more consistently.

## Conclusions

This study found that the handling of commercially identifiable *H. pylori* eradication blister packs had a consistent but modest influence on outpatient AWaRe Access share. Although these packs represented a small proportion of total outpatient antibacterial use, the pack-adjusted and unadjusted series differed consistently across all 33 months, with a median ΔAccess of 0.59 pp.

A transparent and conservative adjustment based on product-name identification and a pack-derived offset applied to both the numerator and denominator provided a pragmatic and reproducible approach for routine surveillance. In this low-Access-share setting, the immediate practical relevance of the pack-related difference is likely to be limited; however, the effect may become increasingly relevant for benchmarking and threshold-based interpretation as prescribing patterns evolve.

The main conclusion of this study is limited to commercially identifiable eradication blister packs, which could be reproducibly identified by product name. Although exploratory analyses suggested that candidate non-pack regimens may further influence Access-share estimates, these analyses had limited specificity and require validation before being used for routine adjustment. Routine surveillance should therefore explicitly define how commercially identifiable eradication packs are handled and, where feasible, use dual reporting of unadjusted and pack-adjusted series to support transparent interpretation.

## Data Availability

The data that support the findings of this study are not publicly available because they consist of anonymized pharmacy dispensing data provided under data-use restrictions, but are available from the corresponding author on reasonable request and with permission of the data provider.

## Abbreviations

AMR: Antimicrobial resistance
AMS: Antimicrobial stewardship
WHO: World Health Organization
AWaRe: Access, Watch, Reserve (The WHO classification system)
ATC: Anatomical Therapeutic Chemical Classification System
J01: ATC group for Systemic Antibacterials
J02: ATC group for Antifungals
J05: ATC group for Antivirals
DDD: Defined daily dose
jDDD: Japanese DDD table
DOT: Days of therapy
pp: percentage points
IQR: Interquartile range
AMPC: Amoxicillin
CAM: Clarithromycin
PPI: Proton pump inhibitor
A02BC: ATC code for Proton Pump Inhibitors
*H. pylori*: *Helicobacter pylori* (The bacterium)
YJ code: Japanese specific pharmaceutical code
